# (*Z*)-Ethyl 3-(2,4-difluoro­anilino)-2-(4-methoxy­phen­yl)acrylate

**DOI:** 10.1107/S1600536808036982

**Published:** 2008-11-20

**Authors:** Zhu-Ping Xiao, He-Ying Xiao

**Affiliations:** a College of Chemistry & Chemical Engineering, Jishou University, Jishou 416000, People’s Republic of China

## Abstract

The title compound, C_18_H_17_F_2_NO_3_, consists of three individually planar subunits, namely two benzene rings and one amino­acrylate group. The amino­acrylate group forms dihedral angles of 5.92 (7) and 50.21 (6)° with the difluoro and methoxy benzene rings, respectively. The dihedral angle between the two benzene rings is 55.25 (7)°. The mol­ecules exhibit intra­molecular N—H⋯O and N—H⋯F inter­actions and form a three-dimensional network *via* inter­molecular C—H⋯O and C—H⋯π hydrogen bonds.

## Related literature

For general background, see: Xiao, Fang *et al.* (2008 ▶); Xiao, Li *et al.* (2008 ▶); Xiao, Xue *et al.* (2007 ▶). For related structures, see: Xiao, Li, Shi *et al.* (2008 ▶); Xiao, Lv *et al.* (2008 ▶); Xiao, Fang *et al.* (2007 ▶).
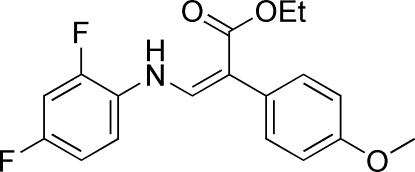


## Experimental

### 

#### Crystal data

C_18_H_17_F_2_NO_3_
*M* *_r_* = 333.33
Monoclinic, 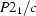

*a* = 17.295 (4) Å
*b* = 7.2940 (15) Å
*c* = 14.233 (3) Å
β = 113.73 (3)°
*V* = 1643.7 (7) Å^3^
*Z* = 4
Mo *K*α radiation
μ = 0.11 mm^−1^
*T* = 298 (2) K
0.30 × 0.20 × 0.10 mm

#### Data collection

Enraf–Nonius CAD-4 diffractometer
Absorption correction: ψ scan (North *et al.*, 1968 ▶) *T*_min_ = 0.969, *T*_max_ = 0.989
3108 measured reflections
2974 independent reflections
1889 reflections with *I* > 2σ(*I*)
*R*_int_ = 0.016

#### Refinement

*R*[*F* ^2^ > 2σ(*F* ^2^)] = 0.053
*wR*(*F* ^2^) = 0.156
*S* = 1.02
2974 reflections
224 parameters
H atoms treated by a mixture of independent and constrained refinement
Δρ_max_ = 0.20 e Å^−3^
Δρ_min_ = −0.18 e Å^−3^

### 

Data collection: *CAD-4 Software* (Enraf–Nonius, 1989 ▶); cell refinement: *CAD-4 Software*; data reduction: *XCAD4* (Harms & Wocadlo, 1995 ▶); program(s) used to solve structure: *SHELXS97* (Sheldrick, 2008 ▶); program(s) used to refine structure: *SHELXL97* (Sheldrick, 2008 ▶); molecular graphics: *SHELXTL* (Sheldrick, 2008 ▶); software used to prepare material for publication: *SHELXL97*.

## Supplementary Material

Crystal structure: contains datablocks global, I. DOI: 10.1107/S1600536808036982/bq2102sup1.cif

Structure factors: contains datablocks I. DOI: 10.1107/S1600536808036982/bq2102Isup2.hkl

Additional supplementary materials:  crystallographic information; 3D view; checkCIF report

## Figures and Tables

**Table 1:** Hydrogen-bond geometry (Å, °)

*D*—H⋯*A*	*D*—H	H⋯*A*	*D*⋯*A*	*D*—H⋯*A*
C6—H6⋯O1^i^	0.93	2.51	3.280 (3)	140
C18—H18*C*⋯*Cg*1^ii^	0.96	2.92	3.631	132
N1—H1⋯F1	0.88 (2)	2.31 (2)	2.678 (2)	105.0 (18)
N1—H1⋯O1	0.88 (2)	2.02 (2)	2.678 (3)	131 (2)

Symmetry codes: (i) 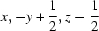; (ii) 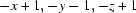. *Cg*1 is the centroid of C7–C12 ring.

**Fig. 1.**
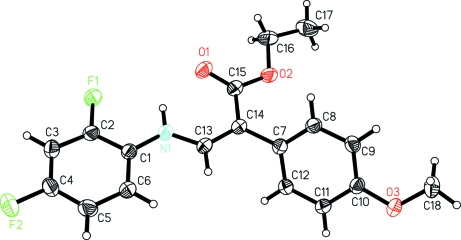
Molecular structure of the title compound, showing the atom-numbering scheme. Displacement ellipsoids are drawn at the 30% probability level. Dashed lines indicate H-bonds.

**Fig. 2.**
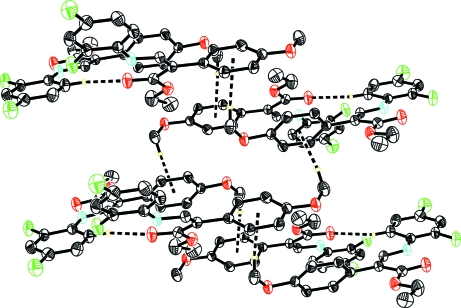
The crystal packing of the title compound. Dashed lines indicate C—H···O and C—H···pi hydrogen bonds.

## supplementary crystallographic information

### Comment

An enamine, a tautomer of a Schiff base, shows a high similarity to the corresponding Schiff base in chemical structure which shows diverse biological activities. Our recent work affirmed that enamine, like Schiff base, exhibited high antibacterial activity (Xiao, Xue *et al.*, 2007; Xiao, Fang *et al.*, 2008; Xiao, Li *et al.*, 2008). Meanwhile, an enamine is the key mediate for anticancer agents, 3-arylquinolone and 3-arylquinoline (Xiao, Li *et al.* 2008; Xiao, Lv *et al.*, 2008; Xiao, Fang *et al.*,2008). We herein report the crystal structure of the title compound, (I), an enamine.

As shown in Fig. 1, (I) is structurally divided into three subunits, and each moiety forms a plane, namely, C1 to C6 forms a plane with the mean deviation of 0.0015 Å, defined as plane I; C7 to C12 forms a plane with the mean deviation of 0.0035 Å, defined as plane II; N1, C13, C14, C15, O1 and O2 is nearly coplanar with the mean deviation of 0.0371 Å, defined as plane III. Plane III make a dihedral angle with plane I and plane II of 5.921 (74) and 50.207 (56) °, while the dihedral angle between plane I and plane II is 55.247 (72) °. The bond distance C13—C14 (1.360 (3) Å) falls in the range of a typical double bond, and C13—N1 bond (1.343 (3) Å) is shorter than the standard C—N single bond (1.48 Å), but longer than a C—N double bond (1.28 Å). This clearly indicates that the p orbital of N1 is conjugated with the π molecular orbital of C13—C14 double bond. All other double bonds and single bonds in the molecule fall in normal range of bond lengths.

The molecule is stabilized by intramolecular interactions N1—H1···O1 and N1—H1···F1 (Table 1), and form one-dimensional infinite chains *via* intermolecular hydrogen bonds C6—H6···O1 (Table 1). These chains are interconnected *via* weak C18—H18C···π (centroid of C7-C12 ring) interactions (Table 1 and Fig. 2).

### Experimental

Equimolar quantities (6 mmol) of ethyl 2-(4-methoxyphenyl)-3- oxopropanoate (1.33 g) and 2,4-difluorobenzenamine (0.77 g) in absolute alcohol (18 ml) were heated at 344–354 K for 1.5 h. The excess solvent was removed under reduced pressure. The residue was purified by a flash chromatography with EtOAc-petrolum ether to afford two fractions. The second fraction gave a E-isomer, and the first fraction, after partial solvent evaporated, furnished colorless blocks of (I) suitable for single-crystal structure determination.

### Refinement

The H atom bonded to N1 was located in a difference Fourier map and refined freely. All other H atoms were placed in geometrically idealized positions and constrained to ride on their parent atoms with C—H = 0.93, 0.96 and 0.97 Å for the aromatic, CH_3_ and CH_2_ type H atoms, respectively. *U*_iso_ = 1.2*U*_eq_(parent atoms) were assigned for amino, aromatic and CH_2_ type H-atoms and 1.5*U*_eq_(parent atoms) for CH_3_ type H-atoms.

### Crystal data

**Table:**

C_18_H_17_F_2_NO_3_	*F*_000_ = 696
*M**_r_* = 333.33	*D*_x_ = 1.347 Mg m^−^^3^
Monoclinic, *P*2_1_/*c*	Mo *K*α radiation λ = 0.71073 Å
Hall symbol: -P 2ybc	Cell parameters from 1729 reflections
*a* = 17.295 (4) Å	θ = 1.4–24.7º
*b* = 7.2940 (15) Å	µ = 0.11 mm^−^^1^
*c* = 14.233 (3) Å	*T* = 298 (2) K
β = 113.73 (3)º	Block, colorless
*V* = 1643.7 (7) Å^3^	0.30 × 0.20 × 0.10 mm
*Z* = 4	

### Data collection

**Table:**

Enraf–Nonius CAD-4 diffractometer	2974 independent reflections
Radiation source: fine-focus sealed tube	1889 reflections with *I* > 2σ(*I*)
Monochromator: graphite	*R*_int_ = 0.016
*T* = 298(2) K	θ_max_ = 25.3º
ω/2θ scans	θ_min_ = 1.3º
Absorption correction: ψ scan(North *et al.*, 1968)	*h* = −20→19
*T*_min_ = 0.969, *T*_max_ = 0.989	*k* = −8→0
3108 measured reflections	*l* = 0→17

### Refinement

**Table:**

Refinement on *F*^2^	Hydrogen site location: inferred from neighbouring sites
Least-squares matrix: full	H atoms treated by a mixture of independent and constrained refinement
*R*[*F*^2^ > 2σ(*F*^2^)] = 0.053	*w* = 1/[σ^2^(*F*_o_^2^) + (0.084*P*)^2^ + 0.0301*P*] where *P* = (*F*_o_^2^ + 2*F*_c_^2^)/3
*wR*(*F*^2^) = 0.156	(Δ/σ)_max_ = 0.003
*S* = 1.02	Δρ_max_ = 0.20 e Å^−^^3^
2974 reflections	Δρ_min_ = −0.18 e Å^−^^3^
224 parameters	Extinction correction: SHELXL97 (Sheldrick, 2008), Fc^*^=kFc[1+0.001xFc^2^λ^3^/sin(2θ)]^-1/4^
Primary atom site location: structure-invariant direct methods	Extinction coefficient: 0.023 (3)
Secondary atom site location: difference Fourier map	

### Special details

**Table:**

Geometry. All e.s.d.'s (except the e.s.d. in the dihedral angle between two l.s. planes) are estimated using the full covariance matrix. The cell e.s.d.'s are taken into account individually in the estimation of e.s.d.'s in distances, angles and torsion angles; correlations between e.s.d.'s in cell parameters are only used when they are defined by crystal symmetry. An approximate (isotropic) treatment of cell e.s.d.'s is used for estimating e.s.d.'s involving l.s. planes.
Refinement. Refinement of *F*^2^ against ALL reflections. The weighted *R*-factor *wR* and goodness of fit *S* are based on *F*^2^, conventional *R*-factors *R* are based on *F*, with *F* set to zero for negative *F*^2^. The threshold expression of *F*^2^ > σ(*F*^2^) is used only for calculating *R*-factors(gt) *etc*. and is not relevant to the choice of reflections for refinement. *R*-factors based on *F*^2^ are statistically about twice as large as those based on *F*, and *R*- factors based on ALL data will be even larger.

### Fractional atomic coordinates and isotropic or equivalent isotropic displacement parameters (Å^2^)

**Table:**

	*x*	*y*	*z*	*U*_iso_*/*U*_eq_	
C1	−0.01149 (13)	0.2255 (3)	0.45020 (18)	0.0442 (6)	
C2	−0.09169 (14)	0.2175 (4)	0.44819 (18)	0.0482 (6)	
C3	−0.16098 (15)	0.1544 (4)	0.3666 (2)	0.0577 (7)	
H3	−0.2143	0.1519	0.3680	0.069*	
C4	−0.14800 (15)	0.0951 (4)	0.28276 (19)	0.0569 (7)	
C5	−0.07067 (16)	0.0977 (4)	0.27953 (19)	0.0601 (7)	
H5	−0.0638	0.0562	0.2216	0.072*	
C6	−0.00210 (15)	0.1628 (4)	0.36312 (18)	0.0536 (7)	
H6	0.0510	0.1647	0.3611	0.064*	
C7	0.28914 (13)	0.3614 (3)	0.64133 (17)	0.0420 (6)	
C8	0.35651 (13)	0.2735 (3)	0.71676 (17)	0.0461 (6)	
H8	0.3477	0.2142	0.7695	0.055*	
C9	0.43614 (14)	0.2706 (4)	0.71668 (18)	0.0492 (6)	
H9	0.4801	0.2104	0.7686	0.059*	
C10	0.45026 (14)	0.3584 (3)	0.63826 (18)	0.0450 (6)	
C11	0.38407 (14)	0.4464 (4)	0.56174 (19)	0.0501 (7)	
H11	0.3928	0.5042	0.5086	0.060*	
C12	0.30493 (14)	0.4487 (3)	0.56390 (17)	0.0472 (6)	
H12	0.2612	0.5101	0.5124	0.057*	
C13	0.13613 (13)	0.3038 (3)	0.55292 (18)	0.0454 (6)	
H13	0.1487	0.2745	0.4970	0.055*	
C14	0.20243 (14)	0.3552 (3)	0.64003 (17)	0.0438 (6)	
C15	0.18698 (15)	0.4010 (3)	0.73038 (19)	0.0486 (6)	
C16	0.24764 (19)	0.4999 (5)	0.9037 (2)	0.0767 (9)	
H16A	0.2256	0.3925	0.9249	0.092*	
H16B	0.2088	0.6007	0.8948	0.092*	
C17	0.3318 (2)	0.5471 (5)	0.9828 (2)	0.0838 (10)	
H17A	0.3708	0.4499	0.9881	0.126*	
H17B	0.3280	0.5633	1.0477	0.126*	
H17C	0.3513	0.6587	0.9639	0.126*	
C18	0.59583 (14)	0.2750 (4)	0.7079 (2)	0.0709 (9)	
H18A	0.6026	0.3184	0.7745	0.106*	
H18B	0.6464	0.2995	0.6975	0.106*	
H18C	0.5854	0.1453	0.7036	0.106*	
F1	−0.10159 (8)	0.2760 (2)	0.53363 (11)	0.0670 (5)	
F2	−0.21570 (10)	0.0299 (3)	0.20048 (12)	0.0843 (6)	
H1	0.0432 (15)	0.311 (4)	0.5919 (19)	0.059 (8)*	
N1	0.05458 (11)	0.2904 (3)	0.53817 (16)	0.0500 (6)	
O1	0.11846 (11)	0.3870 (3)	0.73643 (13)	0.0660 (6)	
O2	0.25571 (10)	0.4636 (3)	0.80903 (12)	0.0558 (5)	
O3	0.52661 (9)	0.3660 (3)	0.63138 (13)	0.0598 (5)	

### Atomic displacement parameters (Å^2^)

**Table:**

	*U*^11^	*U*^22^	*U*^33^	*U*^12^	*U*^13^	*U*^23^
C1	0.0416 (12)	0.0463 (15)	0.0459 (14)	0.0017 (11)	0.0188 (11)	0.0039 (12)
C2	0.0461 (14)	0.0581 (16)	0.0457 (14)	0.0045 (12)	0.0240 (12)	0.0035 (12)
C3	0.0407 (13)	0.0704 (19)	0.0578 (17)	−0.0009 (13)	0.0153 (12)	0.0069 (15)
C4	0.0483 (15)	0.0653 (18)	0.0465 (15)	−0.0029 (13)	0.0080 (12)	0.0009 (13)
C5	0.0618 (17)	0.0738 (19)	0.0476 (15)	0.0055 (15)	0.0249 (13)	−0.0054 (14)
C6	0.0467 (14)	0.0683 (18)	0.0505 (15)	−0.0002 (13)	0.0244 (12)	−0.0037 (14)
C7	0.0416 (13)	0.0416 (14)	0.0442 (13)	−0.0025 (11)	0.0185 (11)	−0.0061 (11)
C8	0.0469 (13)	0.0485 (15)	0.0463 (14)	0.0036 (12)	0.0223 (11)	0.0040 (12)
C9	0.0444 (13)	0.0539 (16)	0.0461 (14)	0.0096 (12)	0.0148 (11)	0.0048 (12)
C10	0.0402 (12)	0.0484 (15)	0.0486 (14)	0.0006 (11)	0.0203 (11)	−0.0049 (12)
C11	0.0473 (14)	0.0597 (17)	0.0478 (14)	0.0012 (12)	0.0236 (12)	0.0077 (13)
C12	0.0416 (13)	0.0531 (16)	0.0449 (14)	0.0037 (12)	0.0155 (11)	0.0054 (12)
C13	0.0429 (13)	0.0489 (15)	0.0497 (14)	0.0010 (11)	0.0240 (11)	−0.0006 (12)
C14	0.0438 (13)	0.0450 (15)	0.0469 (14)	−0.0007 (11)	0.0228 (11)	0.0010 (12)
C15	0.0491 (14)	0.0476 (15)	0.0536 (15)	−0.0027 (12)	0.0253 (13)	0.0020 (12)
C16	0.087 (2)	0.107 (3)	0.0471 (16)	−0.0039 (19)	0.0380 (16)	−0.0040 (17)
C17	0.107 (3)	0.089 (3)	0.0507 (17)	−0.012 (2)	0.0272 (18)	−0.0072 (17)
C18	0.0430 (14)	0.087 (2)	0.078 (2)	0.0110 (15)	0.0204 (14)	0.0032 (18)
F1	0.0506 (8)	0.1011 (13)	0.0573 (9)	0.0033 (8)	0.0300 (7)	−0.0085 (9)
F2	0.0611 (10)	0.1115 (15)	0.0617 (10)	−0.0110 (10)	0.0054 (8)	−0.0135 (10)
N1	0.0404 (11)	0.0651 (15)	0.0487 (13)	−0.0016 (10)	0.0222 (10)	−0.0053 (11)
O1	0.0548 (11)	0.0921 (15)	0.0633 (12)	−0.0091 (10)	0.0366 (9)	−0.0091 (11)
O2	0.0578 (11)	0.0708 (13)	0.0449 (10)	−0.0087 (9)	0.0271 (8)	−0.0088 (9)
O3	0.0412 (9)	0.0763 (13)	0.0671 (12)	0.0043 (9)	0.0274 (9)	0.0062 (10)

### Geometric parameters (Å, °)

**Table:**

C1—C2	1.377 (3)	C11—C12	1.382 (3)
C1—C6	1.390 (3)	C11—H11	0.9300
C1—N1	1.395 (3)	C12—H12	0.9300
C2—F1	1.363 (3)	C13—N1	1.343 (3)
C2—C3	1.370 (3)	C13—C14	1.360 (3)
C3—C4	1.369 (4)	C13—H13	0.9300
C3—H3	0.9300	C14—C15	1.454 (3)
C4—C5	1.357 (3)	C15—O1	1.226 (3)
C4—F2	1.365 (3)	C15—O2	1.343 (3)
C5—C6	1.383 (3)	C16—O2	1.435 (3)
C5—H5	0.9300	C16—C17	1.479 (4)
C6—H6	0.9300	C16—H16A	0.9700
C7—C8	1.384 (3)	C16—H16B	0.9700
C7—C12	1.392 (3)	C17—H17A	0.9600
C7—C14	1.493 (3)	C17—H17B	0.9600
C8—C9	1.378 (3)	C17—H17C	0.9600
C8—H8	0.9300	C18—O3	1.418 (3)
C9—C10	1.391 (3)	C18—H18A	0.9600
C9—H9	0.9300	C18—H18B	0.9600
C10—O3	1.364 (3)	C18—H18C	0.9600
C10—C11	1.380 (3)	N1—H1	0.88 (2)
			
C2—C1—C6	116.5 (2)	C11—C12—H12	119.2
C2—C1—N1	119.2 (2)	C7—C12—H12	119.2
C6—C1—N1	124.4 (2)	N1—C13—C14	127.6 (2)
F1—C2—C3	118.7 (2)	N1—C13—H13	116.2
F1—C2—C1	117.1 (2)	C14—C13—H13	116.2
C3—C2—C1	124.2 (2)	C13—C14—C15	118.8 (2)
C4—C3—C2	116.8 (2)	C13—C14—C7	119.6 (2)
C4—C3—H3	121.6	C15—C14—C7	121.6 (2)
C2—C3—H3	121.6	O1—C15—O2	121.7 (2)
C5—C4—F2	119.5 (2)	O1—C15—C14	124.7 (2)
C5—C4—C3	122.3 (2)	O2—C15—C14	113.5 (2)
F2—C4—C3	118.2 (2)	O2—C16—C17	108.6 (2)
C4—C5—C6	119.4 (2)	O2—C16—H16A	110.0
C4—C5—H5	120.3	C17—C16—H16A	110.0
C6—C5—H5	120.3	O2—C16—H16B	110.0
C5—C6—C1	120.8 (2)	C17—C16—H16B	110.0
C5—C6—H6	119.6	H16A—C16—H16B	108.3
C1—C6—H6	119.6	C16—C17—H17A	109.5
C8—C7—C12	117.0 (2)	C16—C17—H17B	109.5
C8—C7—C14	121.6 (2)	H17A—C17—H17B	109.5
C12—C7—C14	121.3 (2)	C16—C17—H17C	109.5
C9—C8—C7	122.4 (2)	H17A—C17—H17C	109.5
C9—C8—H8	118.8	H17B—C17—H17C	109.5
C7—C8—H8	118.8	O3—C18—H18A	109.5
C8—C9—C10	119.5 (2)	O3—C18—H18B	109.5
C8—C9—H9	120.3	H18A—C18—H18B	109.5
C10—C9—H9	120.3	O3—C18—H18C	109.5
O3—C10—C11	116.4 (2)	H18A—C18—H18C	109.5
O3—C10—C9	124.3 (2)	H18B—C18—H18C	109.5
C11—C10—C9	119.3 (2)	C13—N1—C1	126.5 (2)
C10—C11—C12	120.2 (2)	C13—N1—H1	116.2 (16)
C10—C11—H11	119.9	C1—N1—H1	116.8 (16)
C12—C11—H11	119.9	C15—O2—C16	117.2 (2)
C11—C12—C7	121.6 (2)	C10—O3—C18	117.9 (2)
			
C6—C1—C2—F1	179.1 (2)	C8—C7—C12—C11	0.6 (3)
N1—C1—C2—F1	0.5 (4)	C14—C7—C12—C11	−176.4 (2)
C6—C1—C2—C3	−0.7 (4)	N1—C13—C14—C15	0.6 (4)
N1—C1—C2—C3	−179.2 (2)	N1—C13—C14—C7	179.9 (2)
F1—C2—C3—C4	−179.3 (2)	C8—C7—C14—C13	−128.3 (3)
C1—C2—C3—C4	0.4 (4)	C12—C7—C14—C13	48.6 (3)
C2—C3—C4—C5	0.0 (4)	C8—C7—C14—C15	51.0 (3)
C2—C3—C4—F2	179.5 (2)	C12—C7—C14—C15	−132.1 (2)
F2—C4—C5—C6	−179.7 (2)	C13—C14—C15—O1	4.3 (4)
C3—C4—C5—C6	−0.2 (5)	C7—C14—C15—O1	−174.9 (2)
C4—C5—C6—C1	0.0 (4)	C13—C14—C15—O2	−175.0 (2)
C2—C1—C6—C5	0.4 (4)	C7—C14—C15—O2	5.7 (3)
N1—C1—C6—C5	178.9 (2)	C14—C13—N1—C1	−175.4 (2)
C12—C7—C8—C9	−0.1 (4)	C2—C1—N1—C13	178.8 (2)
C14—C7—C8—C9	177.0 (2)	C6—C1—N1—C13	0.4 (4)
C7—C8—C9—C10	−0.1 (4)	O1—C15—O2—C16	4.9 (4)
C8—C9—C10—O3	179.2 (2)	C14—C15—O2—C16	−175.7 (2)
C8—C9—C10—C11	−0.2 (4)	C17—C16—O2—C15	173.7 (2)
O3—C10—C11—C12	−178.7 (2)	C11—C10—O3—C18	−179.2 (2)
C9—C10—C11—C12	0.7 (4)	C9—C10—O3—C18	1.5 (4)
C10—C11—C12—C7	−1.0 (4)		

### Hydrogen-bond geometry (Å, °)

**Table:**

*D*—H···*A*	*D*—H	H···*A*	*D*···*A*	*D*—H···*A*
C6—H6···O1^i^	0.93	2.51	3.280 (3)	140
C18—H18C···Cg1^ii^	0.96	2.92	3.631	132
N1—H1···F1	0.88 (2)	2.31 (2)	2.678 (2)	105.0 (18)
N1—H1···O1	0.88 (2)	2.02 (2)	2.678 (3)	131 (2)

Symmetry codes: (i) *x*, −*y*+1/2, *z*−1/2; (ii) −*x*+1, −*y*−1, −*z*+1.

## References

[bb1] Enraf–Nonius. (1989). *CAD-4 Software* Enraf–Nonius, Delft, The Netherlands.

[bb2] Harms, K. & Wocadlo, S. (1995). *XCAD4* University of Marburg, Germany.

[bb3] North, A. C. T., Phillips, D. C. & Mathews, F. S. (1968). *Acta Cryst.* A**24**, 351–359.

[bb4] Sheldrick, G. M. (2008). *Acta Cryst.* A**64**, 112–122. 10.1107/S0108767307043930 18156677

[bb5] Xiao, Z.-P., Fang, R.-Q., Li, H.-Q., Shi, L., Xue, J.-Y., Zheng, Y. & Zhu, H.-L. (2008). *Eur. J. Med. Chem.* **43**, 1828–1836. 10.1016/j.ejmech.2007.11.026 18192085

[bb6] Xiao, Z.-P., Fang, R.-Q., Shi, L., Ding, H., Xu, C. & Zhu, H.-L. (2007). *Can. J. Chem.* **85**, 951–957.

[bb7] Xiao, Z.-P., Li, H.-Q., Shi, L., Lv, P.-C., Song, Z.-C. & Zhu, H.-L. (2008). *ChemMedChem*, **3**, 1077–1083. 10.1002/cmdc.200800057 18433075

[bb8] Xiao, Z.-P., Li, H.-Q., Xue, J.-Y., Shi, L. & Zhu, H.-L. (2008). *Synth. Commun.* **38**, 525–529.

[bb9] Xiao, Z.-P., Lv, P.-C., Xu, S.-P., Zhu, T.-T. & Zhu, H.-L. (2008). *ChemMedChem*, **3**, 1516–1519. 10.1002/cmdc.200800160 18712735

[bb10] Xiao, Z.-P., Xue, J.-Y., Tan, S.-H., Li, H.-Q. & Zhu, H.-L. (2007). *Bioorg. Med. Chem.* **15**, 4212–4219. 10.1016/j.bmc.2007.03.060 17418583

